# Stereotype Threat and Gender Bias in Internal Medicine Residency: It is Still Hard to be in Charge

**DOI:** 10.1007/s11606-023-08498-5

**Published:** 2023-11-20

**Authors:** Annabel K. Frank, Jackie J. Lin, Sophia Bellin Warren, Justin L. Bullock, Patricia O’Sullivan, Lauren E. Malishchak, Rebecca A. Berman, Maria A. Yialamas, Karen E. Hauer

**Affiliations:** 1 Department of Medicine, University of California, San Francisco, San Francisco, CA USA; 2 School of Medicine, University of California, San Francisco, San Francisco, CA USA; 3 Department of Medicine, MetroHealth Medical Center, Cleveland, OH USA; 4 Department of Medicine, University of Washington, Seattle, WA USA; 5 Department of Medicine, Brigham and Women’s Hospital, Boston, MA USA

**Keywords:** stereotype threat, gender bias, internal medicine residency, women

## Abstract

**Background:**

Despite similar numbers of women and men in internal medicine (IM) residency, women face unique challenges. Stereotype threat is hypothesized to contribute to underrepresentation of women in academic leadership, and exploring how it manifests in residency may provide insight into forces that perpetuate gender disparities.

**Objective:**

To quantify the prevalence of stereotype threat in IM residency and explore experiences contributing to that stereotype threat.

**Design:**

We used a mixed methods study design. First, we surveyed IM residents using the Stereotype Vulnerability Scale (SVS) to screen for stereotype threat. Second, we conducted focus groups with women who scored high on the SVS to understand experiences that led to stereotype threat.

**Participants:**

The survey was sent to all IM residents at University of California, San Francisco (UCSF), in September–November 2019. Focus groups were conducted at UCSF in Spring 2020.

**Approach:**

The survey included an adapted version of the SVS. For focus groups, we developed a focus group guide informed by literature on stereotype threat. We used a thematic approach to data analysis. The mixed methods design enabled us to draw metainferences by integrating the two data sources.

**Key Results:**

Survey response rate was 61% (110/181). Women were significantly more likely than men to have a score indicating stereotype threat vulnerability (77% vs 0%, *p* < 0.001). Four themes from focus groups characterized women’s experiences of gender bias and stereotype threat: gender norm tension, microaggressions and sexual harassment, authority questioned, and support and allyship.

**Conclusions:**

Gender-based stereotype threat is highly prevalent among women IM residents. This phenomenon poses a threat to confidence and ability to execute patient care responsibilities, detracting from well-being and professional development. These findings indicate that, despite robust representation of women in IM training, further attention is needed to address gendered experiences and contributors to women’s vulnerability to stereotype threat.

**Supplementary Information:**

The online version contains supplementary material available at 10.1007/s11606-023-08498-5.

## INTRODUCTION

Women have achieved tremendous success in medical training and careers, and now comprise an increasing percentage of the physician workforce.^1^ Studies across specialties have demonstrated improvements in various patient outcomes such as mortality, readmission, and procedural measures in patients treated by women compared to men physicians.^2–4^ Despite these successes, women continue to face unique challenges and inequities.

Women physicians experience lower sense of belonging, unequal work-life responsibilities, and higher rates of burnout than their male counterparts.^1, 5–9^ Even women in academic leadership positions experience bullying which impacts their performance, sense of inclusion, and desire for continued advancement.^10^ While women faculty hold more leadership positions than in the past, men are more likely to hold clinical affairs and research affairs deanships whereas women are more likely to hold admissions, diversity, and student affairs deanships, which command lower compensation.^11^ Disparities in compensation exist at all ranks and across specialties, even when controlling for clinical volume, publications, and external funding.^12^ Furthermore, troubling descriptions of sexual harassment and exclusionary culture persist, more commonly described in surgical specialties.^7, 13, 14^ In specialties with greater gender parity such as internal medicine (IM), where in academic year 2021–2022 women accounted for 47% of residents,^15^ it may be assumed the culture is favorable for women. However, descriptions of inequity and bias persist. Gender bias in IM residency, encountered from both patients and other providers, has been shown to pose a threat to fair assessment, clinical learning, and leadership development.^16–18^

To understand impediments to women physicians’ career success, we can look towards theoretical frameworks about how women’s identities shape their experiences. Feminist scholars advocate that, to inform social change, we need to examine the power structures and intersectional forces that underly social injustices.^19, 20^ A feminist theoretical approach demands examination of the cultural context and lived experiences of women. One theoretical framework critical to understanding women’s experiences is stereotype threat. Stereotype threat is a phenomenon in which performance is impaired due to fear of confirming negative stereotypes, and women in historically male-dominated fields, such as math, are particularly susceptible.^21^ Stereotype threat illuminates how women’s confidence and performance are influenced by gender stereotypes, and scholars have hypothesized it may contribute to underrepresentation of women in academic leadership positions.^22^

Survey assessment has shown that women in academic medicine are vulnerable to stereotype threat;^6^ however, no studies have explored the experiences that promote this stereotype threat in IM training. Through this mixed methods study, we sought to understand the experiences of gender bias in IM residency and how these experiences perpetuate stereotype threat vulnerability. The objective of this mixed methods study was to identify women IM residents experiencing stereotype threat based on quantitative data and explore their experiences through qualitative data.

## METHODS

### Design

Using an interpretivist research paradigm, we conducted mixed methods research employing an explanatory sequential design with two phases.^23, 24^ This methodology enables understanding the prevalence of stereotype threat and exploring the concept in greater depth through qualitative data collection specifically with those who experienced stereotype threat. In phase 1, we administered a quantitative survey to assess for stereotype threat and inform our sampling for phase 2. In phase 2, we recruited women who endorsed stereotype threat in the survey. We used focus groups to stimulate discussion about cultural phenomena and enable participants to compare experiences. We chose a general thematic approach to address the research question around women experiencing stereotype threat and gender bias during residency training.^25^

### Team Composition

The study team included eight investigators who identify as women and one man. Three team members are IM clinical faculty with experience in program leadership and education scholarship and one is a faculty leader dedicated to education scholarship. Throughout the project, we had team members at several stages of training including three IM residents who went on to fellowship, one medical student, and one chief resident.

### Setting and Participants

The study was conducted at University of California, San Francisco (UCSF). We surveyed all IM residents at UCSF between September and November 2019. We invited survey respondents who endorsed stereotype threat, defined as a score > 18, to participate in focus groups.^21, 26^ Focus group participants were compensated $25. The UCSF institutional review board approved the study (IRB #19–28,224).

### Phase 1: Quantitative Survey

All UCSF IM residents received email invitations to a survey (Appendix 1) with questions about stereotype threat adapted from the Stereotype Vulnerability Scale (SVS), a survey tool initially developed to measure stereotype threat among women in math classes.^27^ In a recent study, the SVS was adapted for medical students to assess students’ perceptions of stereotype against their race as predictor of vulnerability to stereotype threat.^26^ We similarly adapted the SVS by specifying residency as the context and substituting medical ability for math ability. Additional survey questions included demographic characteristics: assigned gender at birth, gender identity, sexual orientation, race/ethnicity, and age. We chose to survey all residents to compare responses among genders.

We calculated descriptive statistics for all demographics. The SVS score was calculated by summing points from each of the six items with 1 (strongly disagree) to 5 (strongly agree). Two items were reverse-coded so that a higher score indicated increased vulnerability to stereotype threat. SVS score could range from 6 to 30. We characterized SVS scores > 18 as a positive screen for vulnerability to stereotype threat based on prior implementation of the SVS.^26^ A two-tailed Fisher exact test was used to compare the proportion of women versus men who scored positive for stereotype threat.

### Phase 2: Focus Groups

Three investigators (A.K.F., J.L.B., K.E.H.) developed a semi-structured focus group guide (Appendix 2) informed by best practices for focus group development and literature on stereotype threat and gender disparities in medicine.^5, 6, 17, 28, 29^ A.K.F. conducted one pilot focus group in-person with seven recently graduated IM residents including several chief residents. The pilot focus group yielded insight into potential themes which informed probing questions for subsequent focus groups. Pilot participants were eligible for and agreed to participate in the study, and investigators included the pilot in the analysis. A.K.F. conducted two additional focus groups via videoconference platform. We audio-recorded and transcribed all focus groups’ verbatim and deidentified transcripts before analysis. These three focus groups achieved sufficiency using conceptual depth criteria based on the range of clinical experiences and interactions described by participants that informed development of themes: investigators’ identification of a range of exemplars of stereotype threat and gendered experiences that they compared, contrasted, and discussed to refine themes; and resonance of participants’ stories and our themes with literature on gendered experiences.^30^

We used thematic analysis.^31^ A.K.F. reviewed all three transcripts to develop a preliminary codebook. Three investigators (A.K.F., S.B.W., K.E.H.) used the draft codebook to code a transcript individually. After discussion, A.K.F and K.E.H. refined the codebook for clarity and completeness. Using the finalized codebook, two investigators coded each transcript; A.K.F. coded all transcripts and one other investigator coded each transcript (J.J.L., S.B.W., K.E.H.). We reconciled differences in coding through discussion. Interviews were coded using Microsoft Word and uploaded into Dedoose for further analysis.^32^

Five investigators (A.K.F., J.J.L., J.L.B., P.O’S., K.E.H.) reviewed excerpts grouped by code and summarized themes. Investigators iteratively reviewed excerpts grouped by code and discussed the findings to ensure we sufficiently captured the essential themes and variations in perspective.^30^

### Reflexivity

We considered reflexivity throughout the analysis by discussing and documenting our reactions and emotions to the data informed by our personal experiences and perspectives.^33^ Team members at different stages of their careers shared how the data resonated with them and reflected on how gender bias manifests at each stage.

### Data Integration and Metainferences

Two essential characteristics of mixed methods research are integration of the two data sources and drawing metainferences from the integration.^23, 34^ We used survey results to inform questions and probes in the focus groups. We drew insights through analysis of survey and focus group data, and these metainferences are summarized in the results and elaborated in the discussion.

## RESULTS

### Phase 1: Quantitative Survey Assessing Stereotype Threat

The survey response rate was 61% (110/181). Of the respondents, 64 identified as women, 1 as non-binary, and 45 as men. We limited statistical analyses to participants who identified as women or men due to sample size limitations; of note, the non-binary individual did screen positive for stereotype threat (SVS score > 18). Women were significantly more likely than men to have a score indicating experiencing gender-based stereotype threat (77% vs 0%, *p* < 0.001).

### Phase 2: Focus Groups

Of the 110 survey respondents, 39 offered to participate in focus groups. Thirteen were ineligible because they were men or non-binary, and 6 were ineligible because they screened negative for vulnerability to stereotype threat. We recruited from the 20 willing and eligible survey respondents and conducted 2 focus groups with 7 and 3 participants. The pilot focus group included 7 recently graduated residents. In total, 17 women participated in 3 focus groups. We identified 4 themes describing women’s experiences of gender bias and impacting vulnerability to stereotype threat: gender norm tension, microaggressions and sexual harassment, authority questioned, and support and allyship.

### Gender Norm Tension

Residents described gender norm tensions while leading teams, making clinical decisions, and giving orders to nurses and other staff. They felt that societal expectations of their behavior as women were at odds with requirements for successfully performing their job. Participants universally described challenges to fulfill stereotypically feminine approaches of being collaborative and friendly, while still being “*decisive and authoritative*” (FG3-7). One participant described “*I get feedback, I’m not assertive enough….so then I’m more assertive. And then I get feedback that I’m being confrontational…so then I’m way less assertive. And then I get feedback again that I smile too much and that I am not assertive enough. And so it’s impossible to strike this perfect balance*” (FG3-1). This type of negative feedback reinforced to participants “*how thin of a tight rope you are traversing*” (FG2-3) and they felt “penalized” (FG3-4) when attempting to be authoritative.

Such gender norm tension also manifested in participants’ descriptions of the differing expectations of them compared to men peers: “*the expectation is that you always have to be emotionally present as a woman, and when you’re not, it’s like you’re failing. And then for our male colleagues, the expectation is not that they always need to be emotionally present. So when they aren’t, that’s fine…when they are, it’s extra amazing*” (FG2-3). Another participant described how men colleagues received more credit, especially with regard to stereotypically feminine qualities: “*everyone’s like, ‘Wow, he’s a really good listener…People are impressed with him for listening instead of just expecting it like they would of you*” (FG3-2). These multifaceted expectations consumed mental energy: “*exhausting…to be in that space where you’re like, am I nice enough? Am I supportive enough? Am assertive enough? Am I all these things enough*?” (FG2-3). These feelings led women to conclude that they were held to higher standards than men, with wide-ranging and conflicting expectations for their behavior.

Nonetheless, many participants took pride in the perception that they brought valuable interpersonal skills and high emotional intelligence. One participant shared her ability to “*empower different members of the team and bring in everyone’s skills*” (FG2-2). Still, they felt when their communication and collaborative skills shined too much, their clinical strengths would go underrecognized. One participant recalled getting feedback from an attending, “*‘What a great job you do bringing snacks for the team’…I was so offended…write something about my clinical acumen*” (FG2-2). Despite taking pride in their emotional strengths, altogether participants felt burdened by the need to be both warm and authoritative simultaneously, in an environment in which other people seemed to prioritize their warmth and judge them differently than men’s.

### Microaggressions and Sexual Harassment

Participants experienced frequent gender-based microaggressions from other care providers and patients, and overt sexual harassment from patients. These experiences caused participants to doubt their own potential. A common form of microaggression was being misidentified as non-physicians and “*incessantly being called nurse*” (FG1-4). Microaggressions threatened participants’ sense of worth and capability as physicians: “*it gets reinforced, these notions that, ‘Oh, you’re not supposed to do this.’ Because, ‘Oh, are you a nurse?’ Or, ‘Are you sure you’re a doctor? Have you graduated high school yet?’*” (FG3-2). These constant assumptions that participants were non-physicians seeded doubt in their abilities, promoting vulnerability to stereotype threat.

Participants recalled distracting, derogatory comments regarding their speech and appearance from other physicians and patients. One participant recalled “*I was doing an echo on a patient. The whole time, he’s like, ‘… You’re so pretty. I’m so glad I get to watch you while you do this echo’…completely degrades my position and my professional place…I’m just doing the echo and getting sexually harassed the whole time*” (FG3-1). While the constant microaggressions eroded participants’ confidence, overtly sexist incidents stood out in their intensity: “*those big mega experiences, that shook my confidence for months afterwards and still makes me afraid for when I’m going to be in that situation again*”* (*FG3-5*).* Another participant worried about the impact on learning: “*If you’re being harassed by a patient, what is the chance that you’re actually going to remember any of the clinical knowledge that happened in that encounter?… Not a lot of memory forming when the cortisol is coursing through you*” (FG3-7). Altogether, microaggressions and sexual assault made participants feel they did not belong in the physician role, detracted from their learning, and caused self-doubt.

### Authority Questioned

Participants reported that patients, other physicians, and other care providers regularly questioned their authority as resident physicians. Lacking authority made it difficult to carry out their responsibilities, particularly in high-acuity clinical situations. Multiple participants described leading code blues as particularly challenging: “*I was doing it in the way that you’re supposed to, which is speak loudly and clearly, and make clarifications…the feedback I was getting was, ‘Don’t you dare tell me what to push. We’ve been running codes for 20 years. We know how to do this. Don’t you dare tell us how to do compressions’…later, I found out that the nurses who were on that night had given feedback to the ICU supervisor ‘The code leader had been very bossy’*” (FG3-5). One participant summarized, “*when you know you’re supposed to be in charge and there’s no ambiguity, it’s still hard to be in charge*” (FG3-4). Another participant described being interrupted and ignored while trying to give instructions to a team of nurses during a rapid response: “*[I] felt… what am I doing wrong here as a leader that I can’t get this done?…I still struggle with that a little bit….every time I get that feedback or those types of interactions where my role is questioned, it shakes my confidence a little bit. Same with patients, when patients question it too, I’m like, do you really want me as your doctor?*” (FG2-1). Doubt from patients about their competence also negatively affected participants, including one who reported hearing: “*You seem very nice, but you really seem like you don’t know what you’re doing*” (FG2-2). Participants’ experiences with lack of authority across multiple contexts diminished their confidence and led them to question their ability to perform their job, though all felt retrospectively that they were capable.

Because participants felt it was difficult to gain respect, some avoided situations that made them vulnerable, even at the expense of missing learning opportunities. Although a common physician practice at all levels is to run questions by colleagues, participants reported that engaging in this practice could prompt others to assume they were insufficiently competent. One resident described asking a colleague to check a patient with her: “*I’d been managing this patient the whole night and then had done a lot of good initial triaging and management, afterwards I left the room feeling like I had completely failed in a way…the way that this backup person came in…quite condescending, being like well, did you think about this? Should we do this too?*” (FG2-3*).* Similarly, participants hesitated to ask questions because it undermined their authority: “*asking questions, it’s an invitation for someone to mansplain to me essentially*” (FG2-2). Participants deliberately avoided asking questions and considered this to have a direct negative impact on their learning. Even as senior team members, they had difficulty earning respect from interns, colleagues, and attendings: “*One of my male co-residents was like, ‘No, that’s absolutely wrong. This person is in a different type of shock’…. then the attending was like, ‘Oh, yeah. That’s not right.’…then we went into the room and did the exam all together. It was clear that my assessment was correct…[I] had been totally shut down in front of the entire 20 people there, and then actually I was right and no one bothered to say a damn thing*” (FG3-3). These dynamics led participants to feel they needed to prove themselves through double checking and gathering evidence to be sure they were right before speaking up, or not speak at all.

Participants described ways of trying to exude authority. Many opted to wear a white coat and display their badges prominently: “*wear that badge that says, ‘DOCTOR’, in all capital letters like a shield*” (FG2-2). Participants ruminated about how to modify their appearance to gain respect and resented that they had to consider these matters: “*if I look more put together, will people respect me more or less?*” (FG3-3). Nonetheless, any authority they did have felt tenuous and rumination about how to establish authority consumed mental energy.

### Support and Allyship

Participants related acts of support and allyship that helped overcome the consequences of gender bias and stereotype threat. One ICU attending pre-emptively acknowledged that men tend to crowd out women when viewing x-rays, encouraging men to step back and women to step up. Attendings also exhibited allyship by affirming clinical decisions or standing behind the participant so the team would address her during rounds. Participants recalled that both men and women displayed acts of allyship, though women tended to recognize and acknowledge microaggressions more frequently. Many participants described interactions in which no one responded in the moment but later acknowledged that gender bias had occurred; participants appreciated that they noticed but also highlighted this missed opportunity to educate the team in the moment.

Though support from colleagues was appreciated, some participants lamented that they did not have a stronger internal sense of worth. When seeking jobs and networking opportunities, they hesitated more than they observed their male colleagues doing: “*it wasn’t until my husband, who’s a man, was like, ‘Your worth is here, and you’re asking for things here’…. I feel like it’s unfortunate that it takes a man to remind me that I’m worth more than I think I am*” (FG1-1). Participants appreciated when mentors, often women, shared their own struggles with confidence and offered encouragement. Although support in the form of allyship and mentorship helped participants cope with gender bias, they remained vulnerable to stereotype threat in an environment where the threats to confidence were frequent.

## DISCUSSION

Our survey demonstrated that women IM residents are highly vulnerable to stereotype threat and focus group data elucidated the mechanisms and impacts of this stereotype threat vulnerability. Stereotype threat affected self-esteem and performance through gender norm tension, microaggressions and sexual harassment, and questioning women’s authority. While numeric representation of women and men in IM may suggest progress towards gender equity, our findings reveal that significant inequities persist.

We identified two metainferences encapsulating findings from our mixed methods study. First, gender expectations contradicted and therefore diminished women residents’ capacity to be authoritative. Second, because others questioned their abilities and showed disrespect, women residents questioned their own capability. They ruminated over how to change perceptions of them, which detracted from honing their medical competency. Other qualitative studies have similarly shown that women residents had precarious relationships giving orders to nurses, particularly in high-acuity situations, and had trouble with how they were perceived when being assertive, which inhibited their ability to influence patient care.^18, 35, 36^ Data show women emergency medicine residents are rated lower across milestones, with particular gender differences found in procedures, airway management, and emergency stabilization.^29, 37^ A simulation study showed that when female and male actors led the same scripted code blue scenario, the male code leader was rated more highly in communication and leadership skills.^38^ These findings alongside our data support the notion that simply being a woman in appearance, voice, and demeanor is enough to make that physician appear less of a leader in high-acuity medical scenarios.

Our data demonstrate that gender dynamics in residency had immense impact on confidence and cognitive load, which is a concern because these are mechanisms by which stereotype threat can impair performance and growth.^39^ Though the participants in our study were succeeding, as evidenced by their status as women physicians and multiple having been selected as chief residents at a competitive residency program, their experiences of stereotype threat are concerning. They face substantial external and internalized obstacles every day. Participants described frequent microaggressions and sexual harassment; we grouped these together in our results as offenses existing along a continuum of frequent minor insults to infrequent, high-severity incidents.^40^ These events take a toll on the affected residents, who often do not see a mechanism to report these events or potential benefit of doing so.^41^ Though it may seem that these women have “made it,” our findings indicate there remains painful inequity.

Creating solutions to gender bias and the stereotype threat it perpetuates is challenging because of unconscious bias and external as well as internalized stereotypes. Women in our focus groups identified factors that helped them persevere: (1) role models and mentors who genuinely believed in them and pushed them, and (2) gaining experience, knowledge, and confidence in their abilities despite external skepticism. Prior literature supports the notion that role models have an important role in mitigating stereotype threat and improving women’s performance, for example, in mathematics.^42–44^ In addition to interactions with salient role models, several other interventions show promise for reducing stereotype threat, including education on bias and stereotype threat, self-affirmations, and promoting growth mindsets.^22, 45–50^ In a randomized study, a “gender bias habit-changing” workshop improved faculty awareness of gender bias and changed their behaviors to improve the climate for women in intervention departments and not in the control departments.^45^ Importantly, stereotype threat can be reduced and academic performance improved when learners are encouraged to view intelligence as malleable rather than fixed.^47, 48^ Accordingly, women physicians should be encouraged to view leadership skills as teachable and attainable. Chronic stereotype threat can lead to domain disidentification which can manifest as abandonment of ambition.^51^ The goal of any intervention should be to reduce stereotype threat and prevent women residents from doubting their potential as leaders, a concerning possibility raised by our study and similar research,^18, 35, 37^ and a viewpoint that will make it hard for women to close leadership gaps in academic medicine.

Limitations to our study include that women who are most impacted by gender bias and vulnerable to stereotype threat may have been more likely to complete the survey and join focus groups. The number of focus group participants was modest. We were not able to explore intersectionality. The study was not designed to explore experiences of gender diverse trainees, including but not limited to transmen, transwomen, non-binary, or genderfluid individuals. Given our respondent pool, with a single non-binary participant, our team balanced the intended focus of the study, the risk of epistemic violence via exclusion of gender diverse people, and the risk of overgeneralizing the experience of a single individual.

## CONCLUSIONS

This study demonstrates that women in IM residency are highly vulnerable to stereotype threat. The mixed methods design enabled us to elucidate the mechanisms by which stereotype threat arises and persists. Women are treated with a lack of authority and made to feel unfit for the physician role, impacting their confidence and efficacy. In addition to cultural efforts to reduce gender stereotypes and workplace bias, targeted interventions should be undertaken to alleviate resident vulnerability to stereotype threat.

### Supplementary Information

Below is the link to the electronic supplementary material.

Supplementary file1 (DOCX 26 KB)
