# Supplementary material for: Stereotype Threat and Gender Bias in Internal Medicine Residency: It is Still Hard to be in Charge
Source: J Gen Intern Med. 2023 Nov 20;39(4):636–42. doi: 10.1007/s11606-023-08498-5 (PMC10973325; doi:10.1007/s11606-023-08498-5)
Supplement: Supplementary file 1 — Supplementary file1 (DOCX 26 KB) [file 11606_2023_8498_MOESM1_ESM.docx]

**Appendix 1**. Survey

We are conducting a study about the impact of gender stereotypes in residency. There are no right or wrong answers to the following statements; we are interested in your honest reactions and opinions. For the purpose of these statements, others/people refers to anyone with whom you interact at work including patients, nurses, respiratory therapists, social workers, students, attendings, fellows, other IM residents, residents of other specialties, etc.

|  | Strongly disagree (1) | Disagree (2) | Neither agree nor disagree (3) | Somewhat agree (4) | Strongly agree (5) |
| --- | --- | --- | --- | --- | --- |
| Some people expect me to do poorly in residency because of my gender. (1) |  |  |  |  |  |
| Residency may be easier for people of my gender. (2) |  |  |  |  |  |
| Some people feel I have less medical ability because of my gender. (3) |  |  |  |  |  |
| In residency, people of my gender often face biased evaluations or feedback from others. (4) |  |  |  |  |  |
| My gender does not affect people’s perception of my medical ability. (5) |  |  |  |  |  |
| In residency, I often feel that others look down on me because of my gender. (6) |  |  |  |  |  |

Q2 Which term do you use to describe your current gender identity?

- Female
- Male
- Transgender female
- Transgender male
- Gender nonconforming
- Non-binary
- Other__________________________________________________
- Prefer not to answer

Q3 What was your sex assigned at birth?

- Female
- Male
- Intersex

Q4 How would you describe your sexual orientation? Choose all that apply.

- Asexual
- Bisexual
- Gay or lesbian
- Straight/heterosexual
- Queer
- Questioning or unsure
- Other
- Prefer not to answer

Q5 What is your age?

▼ < 20 (1) ... 45 (26)

Q6 What is your racial or ethnic identification? Choose all that apply.

- Asian
- Black
- Hispanic/Latinx
- Native American
- Pacific Islander
- White
- Other
- Prefer not to answer

Q7 We will invite some residents who complete this survey to share their perspectives in a focus group. You would receive $25 compensation for your participation in a focus group. If you are interested, please enter your email address below.

________________________________________________________________

**Appendix 2**. Focus group guide

Introductory questions

*Written exercise—participants can also use the paper later to jot down thoughts during discussion*

We are all women professionals in the field of medicine. When you think about your experience as a woman professional, what comes to mind?

Transition questions

There are some human qualities that some people consider characteristically feminine.

How do you think these qualities may help you excel as a resident physician?

Are there any ways that these feminine qualities are unhelpful for being a resident?

Probe—similarly, can you think of some characteristically masculine qualities, and how these might help residents excel? Are there any ways that these masculine qualities are unhelpful?

Probes:

*Tell me more about that / Can you say more about that? Does anyone have thoughts to add?*

*Do any of you have a similar/different experience/perspective?*

*Emotions: Tell me about how that made you feel / Can you share a specific time you felt that way?*

*Allyship: How did others react? What would have been helpful to you? (cultural shifts, systematic changes?) What did you do / do you do to overcome these challenges? (mentor, colleagues, behavioral change exploration, being willing to ‘wing it’)*

Intro and transition questions ~25 min

Key questions

1. Tell us about a time you felt someone **doubted your clinical capability** because of your gender.
   1. How did that impact your performance/ability to care for patients and growth/learning on that rotation?
   2. Have you received feedback that you felt was influenced by your gender?
2. In what way does gender stereotyping impact your **confidence in yourself**?
   1. In comparison to your male colleagues, do you feel you have more or less confidence in yourself? What makes you think that?
   2. Are there particular areas related to clinical practice or medical careers where you feel you have more or less confidence than your male colleagues? Can you tell us about that?
3. Do you think that being a woman affects your **performance in residency**?
   1. How do you think that will affect your career?
   2. Has it affected your ability to **meet your potential** in residency?
      1. Potential compared to what you expected or compared to your male colleagues?
      2. Probe about how performance and growth is affected by heightened awareness of stereotypes, cognitive load, incongruent expectations, confidence, specific competencies that have been hard to achieve

**Ending question**

1. I wanted to leave some time at the end to give you the opportunity to share anything you’d like to add.
   1. Are there other important aspects of your experience being a woman in residency training that you’d like to share?
   2. Is there anything you’d like to go back to discuss further?
2. Do you have any ideas about how we can minimize these challenges for female residents?

**Summary question** (after short 2–3-min oral summary)

1. Did I correctly capture what was said?

**Final question**

1. Is there anything we should have talked about but didn’t?
